# The Palestinian health research system: who orchestrates the system, how and based on what? A qualitative assessment

**DOI:** 10.1186/s12961-018-0347-4

**Published:** 2018-07-31

**Authors:** Mohammed AlKhaldi, Abdulsalam Alkaiyat, Yehia Abed, Constanze Pfeiffer, Rana Halaseh, Ruba Salah, Manar Idries, Said Abueida, Ibrahim Idries, Ibrahim Jeries, Hamza Meghari, Ali Shaar, Marcel Tanner, Saleem Haj-Yahia

**Affiliations:** 1 Swiss Tropical and Public Health Institute, Socinstr. 57, 4002 Basel, Switzerland; 2 University of Basel, Petersplatz 1, 4003 Basel, Switzerland; 3 Faculty of Public Health, Al Quds University, Jerusalem, Palestine; 4 Cardiovascular Institute, Glasgow University, Glasgow, United Kingdom; 5 Faculty of Medicine and Health Sciences, Najah National University, Nablus, Palestine; 6 School of Clinical Sciences, Bristol University, Bristol, United Kingdom; 7 University College London UCL, London, United Kingdom; 8 United Nations Population Fund, Jerusalem, Palestine

**Keywords:** Health experts, Health research system, Palestine, Stewardship

## Abstract

**Background:**

In 2011, the WHO Eastern Mediterranean Region committee launched a strategy for scaling up research in the region to address the countries’ health needs through formulating and analysing the National Health Research System (HRS). Stewardship comprises three functions, namely governance, policy and priorities, and is a central pillar of this system to ensure a well-organised and functioning HRS. This study aims to examine the perceptions of the HRS performers to understand these functions and to generate insights for system strengthening.

**Methods:**

The study was carried out in Palestine, targetting three sectors in the health field, including relevant governmental health institutions, schools of public health, and major local and international health agencies. The data were collected through 52 in-depth interviews (IDIs) and 6 focus group discussions (FGDs) with policy-makers, academics, directors, and experts. Participants and institutions were selected purposively based on a set of criteria and peer review.

**Results:**

A total of 104 experts participated in the IDIs (52 participants) and FGDs (52 participants in 6 FGDs), highlighting that stewardship functions remain problematic and insufficiently performed, mainly due to a missing health research structural and regulatory framework and dispersed health research work. Despite the limited good practices, the majority of the participants described the Ethical Review and Clearance as weak due to the lack of an agreed-upon national committee and procedural quality and ethics guidelines for non-compliance. A policy or strategy dedicated to health research is lacking. The exercises of research priority-setting appear to be evolving despite the lack of consensus and the low levels of knowledge and experience in research prioritisation. Common gaps, such as weak political will and capacity support, the absence of a national unified regulating body, and the indirect effects of political conditions on strengthening the HRS as well as other sectors, also emerged.

**Conclusions:**

The stewardship functions of the Palestinian HRS remain weak along with substantial political, structural, and resources and capacity gaps. The study emphasises the imperative need to initiate strategic efforts led by the MOH and the Palestinian National Institute of Public Health alongside with other players to strengthen a national HRS through improving the stewardship functions. To achieve this, attention and support of decision-makers, involvement, mobilisation and strategic dialogue are indispensable, in order to embark on building a well-regulated and coordinated structure, operational research policy, and prioritisation of essential research.

**Electronic supplementary material:**

The online version of this article (10.1186/s12961-018-0347-4) contains supplementary material, which is available to authorized users.

## Background

Stewardship and governance are indispensable pillars of health research systems (HRSs), representing two sides of a single coin in the building and development of HRSs. Given growing international concern, this study addresses the aspect of stewardship, wherein functions should be vision driven, well operated, and priority based. The work presented herein forms part of two relevant studies the first of which dealt with the overall understanding of the HRS concepts (AlKhaldi et al., 2018, in press). Herein, the aspect of HRS performance will be analysed. WHO emphasises the importance of research to achieve universal health coverage [1] and focuses on the performance of HRS analysis exercises, including stewardship functions, encompassing governance, policies and prioritisation, to be embedded into HRS [2, 3]. Since health research (HR) often fails to be prioritised, is politically undervalued and poorly organised, WHO has called for a cohesive management based on effective policy and a priority for HR to build national HRSs [4].

Certainly, a successful HRS essentially builds on stewardship, which is a contemporary concept and a model of governance [5–7]. Stewardship is characterised by (1) a regulation and coordination structure with a normative dimension; (2) adopting a clear strategic HR policy; and (3) dynamic priority-setting derived from needs [8]. A strong political will is crucial for the development of a HRS and to make important and sometimes difficult decisions about health improvements [9]. A healthcare system (HCS) is defined as “*The organizations, people, and actions whose primary intent is to promote, restore, and maintain health…*” [10], indicating that governance is one of HCS’s building blocks in the framework of systems thinking. Governance falls under stewardship, which, in turn, is defined as the “*responsible management of the well-being of the population*” [11]. These functions are assumed to be the tasks of policy-makers with the presence of a well-functioning system to generate, adapt and apply HR results to address challenges [12]. The aim of HRS analysis is first to understand its concepts and performance (AlKhaldi et al., 2018, in press) and subsequently its functions and capacity. This will ensure that, based on a strategic vision, the system is well governed and resourced. Governance sub-functions include system vision, structure, policy formulation, priority-setting, monitoring and evaluation, advocacy, and the setting of norms, standards and ethical frameworks [4, 6].

Although it is rarely conducted, conceptualising the role of HCS governance is a valuable necessity [11]. Being poor should not disqualify a country from such conceptualisation, because effective research management gives such countries much stronger responsibility for the essential priorities. HR is not only one of these priorities but also a fundamental pillar for achieving the Sustainable Development Goals [13]. Evidently, political support, governance and resources are essential to enhance system performance [14] as hinted by AlKhaldi et al. (2018, in press). Good practice in research systems is required to aid effectiveness, and understanding the system context and governance capacity is essential for system strengthening [15].

In many developing countries, bad governance, poorly functioning policy and a lack of prioritisation still pose obstacles and remain the weakest pillar of HRSs [7, 16–18]. HRS functions are often not recognised where many of them operate almost in an ‘ad hoc’ way and isolated from other research endeavors [19]. Building HR capacity by understanding these practicalities is imperative to improve HR ethics and quality [20]. Therefore, governance is essential to promote a good HR that complies with ethical guidelines and is relevant to the needs of the society [4].

Donor support for countries to build proper research institutions is often inadequate [5]. This weakness may be at its most extreme in the Middle Eastern Region (MER), where formal HRS and functions are considerably fragmented and uncoordinated. As its concepts are often not understood (AlKhaldi et al., 2018, in press), basic building blocks for HRS, including stewardship, are lacking, alongside a deficit in political pledge [21, 22]. Policies and prioritisation are inadequate due to stakeholder disengagement, data unavailability and capacity constraints [23]. Published HR in the region does not align with stated priorities, and governance represents the main gap in health policy and systems research [24].

This study meets the international calls and regional demands for analysing HRSs, with its results expected to have a positive impact on health and other sectors. Assessments in fragile settings such as Palestine are needed to understand options for strengthening of the HRS [25], which is of a particular national strategic need in Palestine given that it is in the process of being built. Further, there is an urgent need to build a system able to economise resources and improve health. Much like other MER countries, Palestine is facing a real crisis in governance and leadership, mainly due to the Israeli occupation and political instability [26–28]. There are other gaps, such as insufficient resources and strategic planning, inequity and poor quality of care, fragmented information, and other interconnected development challenges [29]. To realistically address these gaps, a responsive, effective, resilient and flexible HRS is required.

Given the shortage of HRS stewardship literature, this study seeks to bridge the knowledge gap by analyising this vital component to generate visions to strengthen it. As a logical progressive step, the study is the third in a larger investigation that aims to examine the Palestinian HRS in order to achieve a comprehensive and system understanding. The study intends to investigate the landscape of stewardship functions and recognise the relevant gaps by exploring the status of HRS governance, policy and priority-setting. This study examines the perceptions of relevant health experts to realise the following objectives:

1. Investigate the current governance framework related to HRS management structure and stakeholders’ practices, coordination and cooperation (C&C) mechanisms, and HR ethical review and clearance (ERC) processes.
2. Assess HRS capacity in terms of strategy and National HR policy (NHRP) in terms of availability, formulation and implementation.
3. Evaluate HR priority-setting and its alignment to the actual and actively identified national health needs, and accordingly generate useful prospects for a strengthened HRS stewardship, integrating its three functions of governance, HR policy and priorities.

## Methods

**Fig. 1:**
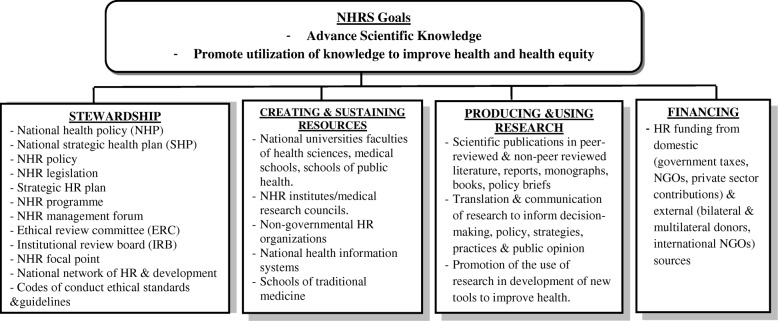
NHRS conceptual framework adapted from Pang et al. [30]

The study’s approach applied the methods and setting of the other studies in the series (AlKhaldi et al., 2018, in press). System analysis frameworks were used, mainly the framework according to Pang et al. [30], as illustrated in Fig. 1, together with other approaches such as system thinking and comprehensive HRS assessment [2, 6, 10, 16, 30]. These approaches help to provide the groundwork for system improvement and contribute to a better understanding of the subject from different perspectives [31]. The participating institutions’ profile across, government, academia, and the local and international non-governmental organisations (NGOs), inclusion and exclusion selection criteria as well as the study tools were similar to the study of AlKhaldi et al. (2018, in press). The study setting was in Palestine, West Bank (WB) and Gaza Strip (GS), and ran from January until July 2016. Two qualitative methods, namely in-depth interviews (IDIs) and focus group discussions (FGDs), were used to inductively assess the perceptions on the stewardship functions based on different system analysis frameworks [2, 4, 16, 31, 32].

Diverse participants have been purposefully selected equally from both sites in WB and GS based on advance knowledge and experts’ consultations. In order to attain adequate information, participation and representation, criterion sampling, critical case, snowball and homogeneous sampling were performed [33]. A total of 52 IDIs, lasting on average 45 minutes and 6 sectorial FGDs were conducted with 52 participants for 1 hour and a half on average. Data collection was performed by a research-trained team and supervised by the principal investigator. Data were audio-recorded in the native language Arabic, translated into English and transcribed into MS word sheets at the same time, precisely revised, checked and cleaned for accuracy. Thematic and content approaches were applied using MAXQDA 12 (VERBI GmbH, Berlin), a software package for qualitative data management and analysis. All these procedures, along with data revision and coding for IDIs and FGDs, were performed by the principal investigator.

## Results

### Sociodemographic characteristics of participants

As described elsewhere (AlKhaldi et al., 2018, in press), of the 115 experts from 38 institutions across three sectors invited to participate, 104 agreed and actively responded to both methods of inquiry, while 11 persons declined due to scheduling conflicts. As HR is conceptually broad [34], participants came from diverse backgrounds, expertise and public health disciplines.

### The status of Palestinian HRS governance

Based on the perceptions obtained from the participants and from the IDIs and FGDs, our findings covered the the aspects of overall governance landscape, C&C and ERC.

#### Governance landscape

**Fig. 2:**
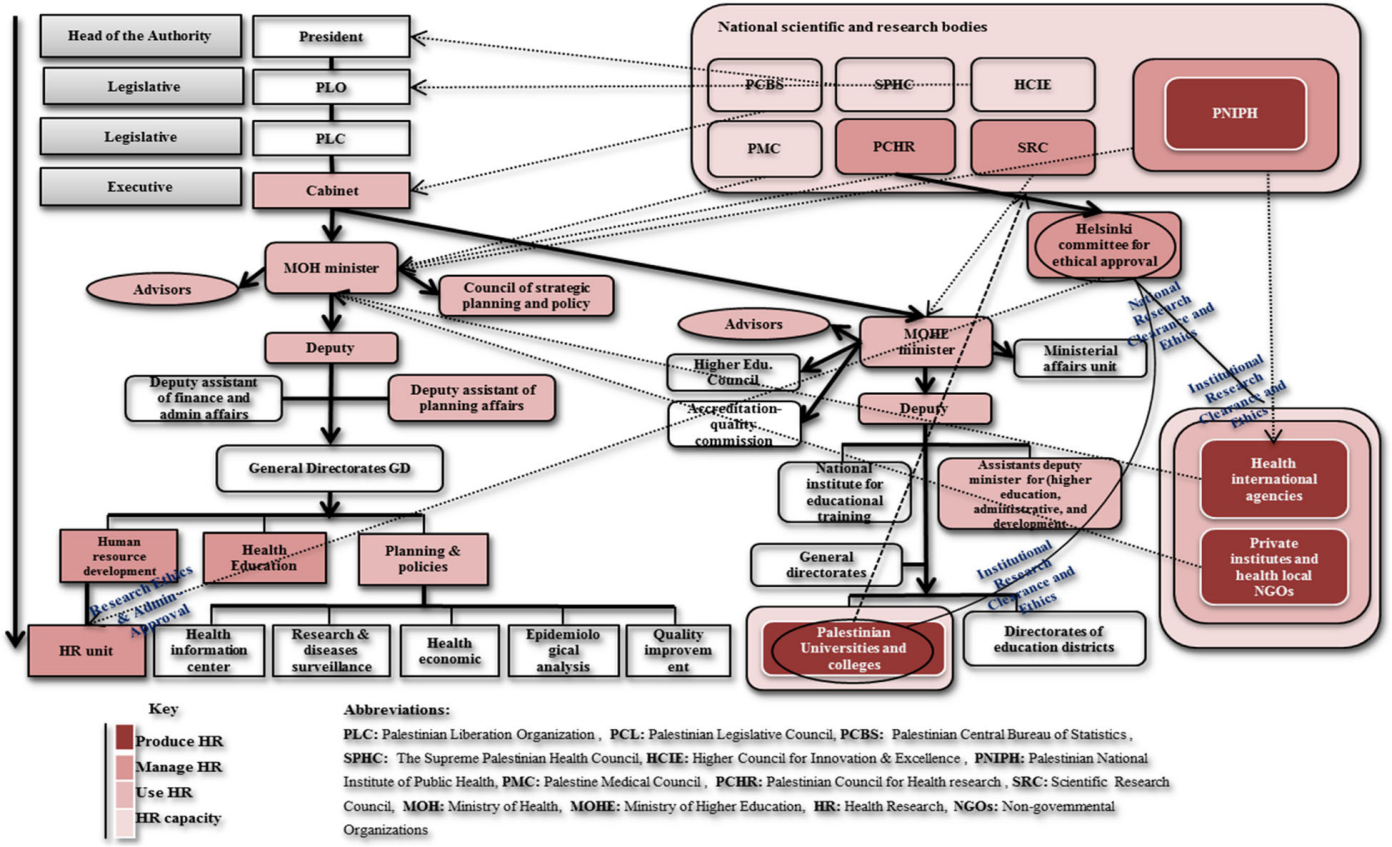
Palestinian health research architecture

The vast majority of participants overwhelmingly agreed that Palestine lacks a clear national governance body; indeed, in the Palestinian national governance structure, HR governance is still fundamentally unstructured and dysfunctional (Fig. 2). The absence of a collective and organised national body is seen as a key problem by a range of experts, with government FGDs attributing this to unconsolidated HR agendas. A former official argued that multiple bodies result in conflicting vision, agenda and scattered efforts. This negatively restricts the contributions of the stakeholders. A government expert clearly echoed:

“*… Actually, there is no good governance body for HRS on the ground, due to a variety of HR entities in Palestine. However, these entities are not functioning well and their entire efforts are not well-coordinated. Most importantly, these bodies do not have a complete HR common vision; all the relevant HRS stakeholders do not work on the same track. This dissipates their contributions and weakens their roles, and mainly affects the performance of health governance and management. Institutionally, we may see a form of HR governance because these institutions have organisational rules and regulation*.” (Gov. Expert 2)

Further consistent views by a Palestinian Legislative Council member admitted the existence of several HR departments within health institutions; however, a national system linking these departments is absent. This prospective system could play a role in establishing a legal framework if it was supported by the government and Ministry of Health (MOH) leadership. Academics largely shared this view, with one of them stressing that: “*The governance concepts are not ready enough or applied as a system and not adopted as a tool for decision-making, while many attempts have been made to establish a national HR council, most of them have failed*” (Acad. Expert 1).

Structurally, several experts from the three sectors noted that HR is not a core component of the HCS, since this system is neither research oriented nor evidence guided. The expert added that, without HRS, the harmony between all institutions is lost. Moreover, one academic gave a comprehensive view of the governance: “*Each institution is independent, whether it is NGO, academic, or governmental, and each one has its own management. So, we do not have a common policy for all institutions.*” Another academic view contrasted with the overall perception, as this view reflected political reality:

“*… It is difficult to understand the concept of governance under occupation. We could not adopt this concept because we do not have control over resources. Governance is controlled by Israel, they collect our tax money for themselves, and they even control importing and exporting the goods. There is no system, yet there are good individual attempts to collaborate with one another to produce research. The research, which forms policies, does not exist. Priorities are political because we are under occupation. I can name our situation as ‘population in danger’. The GS is an open prison, people are suffering, and they are living a true torture. On the other side, the WB undergoes occupation and threat to the lives of people. I can clearly see that priorities are not in favor of HR for many reasons; first, political instability and disintegration; secondly, lack of salaries and income. We have a structural problem*.” (Acad. Expert 15)

Most NGOs’ perceptions were actually consistent with this view and reflected on the lack of structural governance and policy built into the Palestinian HCS architecture. This perception intersects with views held by government and academic sectors. One of these views was stated by an Officer of the United Nations Relief and Works Agency for Palestine Refugees in the Near East:

“*… HR is organised by the international community. Recently, the Palestinian universities played a role in organising research but their role is still not very robust. This is because most of the HR is done by students, and also it is solicited and controlled by donors. HR is not systematic and not a leadership concern, and not fully integrated into the HCS, which functions separately. In fact, a group of brilliant and qualified academics and professionals are exclusively working in HR in Palestine*.” (International NGO Expert 2)

Several experts held the view that uncertain HRS governance is due to individualism, lack of coordination and competitiveness rather than complementarity. An international NGO expert asserted that “*Efforts made to improve the Palestinian HRS are individualistic and uncoordinated due to the lack of a clear structure to guide the HRS actions*” (International NGO Expert 3). Furthermore, HCS and HRS are currently experiencing an identical challenge, which is ineffective management and improper resources distribution. Many experts revealed the weakness of the MOH’s organising role of HR due to the lack of serious political decision. A variety of FGDs’ views stated that the MOH seems to be perceived only as a care provider with very limited HRS capacity. There was a claim for engaging the MOH and demonstrating transparency in HR policy and practice. Others referred low facilitation in HR activities to the lack of an enabling environment. Many experts, particularly academics, criticised the interference of political conditions and bureaucratic government procedures, which negatively affect the strengthening of the HRS. Three local NGO experts raised this point:

“*… The problem of HR is that we still confront a gap and lack of organisation and communication between the policy-makers and the education sector. There is no national policy that manages the work of HR and we always refer to the MOH as a key player to do this task. I can explain it as due to the lack of priorities and the fact that the MOH’s role is vague, is it a service provider or is it a regulator?*” (Local NGO Expert 5)

Two NGO experts and the academics reflected a range of views. An international NGO expert stated that the government does not invest strategically in education through research because of the small budgets allocated to research. Therefore, due to the weak economic position of the Palestinian government, HR is not a priority. The academics blamed the NGO sector for being preoccupied with other humanitarian projects.

Moreover, some government seniors frequently attributed the absence of an organised system to the fact that HR is controlled by donors, based on short-term projects and consists of a multiplicity of bodies and unclear HR leadership accountability or duty. They revealed that HR does not receive political concern, while the resources and economic constraints make building a unified body difficult. In contrast, one response indicated that HRS is not reflected and institutionalised in the Palestinian HCS structure. A concluding viewpoint was stated by an expert: “*we are currently in a chaos status; scattered initiatives without a united reference body*” (NGOs FGDs).

NGO experts delineated that HRS should not be the individual or unilateral responsibility of a particular party but rather a collective effort synergised among all relevant stakeholders. Governmental and NGO experts voiced that the Palestinian Council for HR (PCHR) had a respectable start in terms of establishing HRS governance and priorities, but this role had markedly declined. Many were not satisfied with this body’s performance, because it existed only nominally and was functionally ineffective. Some NGO experts trusted the Palestinian National Institute of Public Health (PNIPH), while many criticised its current role regarding HRS. The following bodies have been proposed to be able to orchestrate HRS governance activities prospectively.

1. PNIPH, an independent body operated by the government and WHO through a collaboration started in 2013, headquartered in WB with limited presence in GS
2. MOH, particularly Human Resources Departments as a regulator; one department exists in WB and one in GS
3. Major universities as host institutions such as the Institute of Community and Public Health
4. MOH and Palestinian Central Bureau of Statistics jointly
5. PCHR
6. The Supreme Palestinian Health Council

This emphasises that the aspect of who could or does govern and how to build and manage this system, which have been controversial points in the perspectives from all three sectors. Another significant and concise response outlined by an NGO expert summarises these findings: “*as long as we do not have an organising framework, we will remain in a closed circle of chaos regardless of how much coordination we made.*”

Figure 2 portrays the existing national structure of HRS governance and the relationships among the principal involved institutions. The principal investigator designed this illustration based on the experts’ perceptions and realistic depiction. HRS structure seems unclear and hard to be comprehended, where the actors’ tasks, responsibilities and relationships overlap on three levels – national, inter-sectoral and interinstitutional – because of an absence of a national inclusive body, clear strategy, and regulating policy to HR practice.

Additional file 1: Table S1, illustrates the perceptions concerning the common challenges hindering the foundation of a good HR governance system. Challenges were classified into three types: national/structural, prevailing environment and technical. The structural challenges stated were an unconsolidated vision, unclear framework and absence of political reference; a multiplicity of bodies; HRS non-embeddedness into HCS frame and being individualistic; adverse effects of ministerial changes; and centralised and bureaucratic HCS with lack of legal framework. The common environmental challenges detected were mainly political, economic and social pressures; burdens of the occupation; and lack of state sovereignty over resources. The technical challenges were seen as a lack of HR quality, coordination, leadership, supportive environment, accountability, transparency, monitoring and evaluation, qualified staff and resources; HR not a priority, unvalued politically and donor driven and, lastly, HR and evidence-based practice not being embedded in the culture and not well executed. For improving HRS governance, the overall perception suggested building a national HRS comprising a legal and organisational framework under an advisory board. This body should be run by the MOH with international support. The process should be fostered by a robust political will. The main duties of this body would be to formulate an agreed HR vision, build an effective policy, set regular HR priorities and allocate resources, reinforce C&C and organise the stakeholders’ roles. Other key duties would be entrenching HRS concepts, practices and interdisciplinary research. Additional file 1: Table S1 also shows the prospects for speeding up improvements.

#### The status of HRS coordination and cooperation

Additional file 1: Table S2 illustrates the overall reflections about HRS C&C. The majority of experts confirmed that C&C constitutes a major gap echoed in the notions of ‘lack of C&C’, ‘fragile, weak, and fragmented and non-institutionalised’, ‘individualistic-driven’, ‘unsatisfactory’, ‘fluctuated and seasonal’, ‘competitive’ and ‘overlapped’. Some experts described C&C as being one of the weakest HRS components, while a very limited number expressed the existence of good relationships. Some NGO experts echoed that research C&C in the NGOs is well-coordinated without duplication, but uncoordinated at the macro level.

The most stated structural gaps of C&C, characterised by the lack of substantial elements, were a cohesive body, a common vision for an agreed on HR strategy and coordinating plans, mechanisms and policy, the spirit of harmonised teamwork, the existence of state bureaucratic procedures, and communications and partnerships. Other arguments were more technical, notably that HR is externally driven, non-systematically performed based on irrelevant agendas and not on agreed-upon HR priorities, duplication of activities, lack of resources and awareness on HR, mistrust of institutions, disconnection between policy-making and researchers, as well as difficulties of knowledge and data dissemination and accessibility. The final gaps were political problems resulting from the occupation alongside the intra-political division; these problems led to a significant decline in national and institutional relations and C&C. Experts suggested the following ways and means to improve C&C:

1. Advancing PNIPH capacity or developing a collective HRS body with an advisory board
2. Investing in developing consolidated C&C mechanisms by using technology and a platform such as the Lancet Palestinian Health Alliance (LPHA)
3. Launching serious policy dialogues to develop agreed HR agendas geared nationally by forming a joint priorities’ committee including the MOH, PNIPH, academia and NGOs
4. Forming real partnerships to build HRS capacities with division of stakeholders’ roles
5. Promoting incentives, resources, HR culture, teamwork and multidisciplinarity
6. Establishing a reference commission between policy-making and research people

#### Ethical review and clearance (ERC) of HRS in Palestine

Additional file 1: Table S3 displays the selected perceptions from three sectors addressing the ERC. Most of the views revealed a major weakness in the ERC, which was described as not well regulated. Some stated that it is unstructured and not performing well due to (1) a governance gap, ERC being just a nominal process, lack of standards, low quality, slow and non-rigorous procedures, and insufficiency of expertise, as well as (2) knowledge limitation concerning ERC outside the institution and a lack of conviction for the good application of research ethics and compliance with international standards.

Diverse perceptions of national and institutional ethics committees were reported. A limited number of experts mentioned the Helsinki ethical approval committee in GS, the only ERC national committee, which manages and examines the HR ethics of submitted research proposals by relevant institutions. Figure 2 shows the main accredited ERC entities placed in the Palestinian HR national structure. The Helsinki committee – one of the key entities – is affiliated to the PCHR. This council is hosted by the MOH and comprises diverse members on its board. The Helsinki committee interconnects with three sectors, mainly the HR unit of the MOH for HR administrative and technical facilitation. Many experts were not pleased with this committee’s performance, and its political and legal reference is still missing and uncertain.

The majority asserted that the Institutional Review Board exists essentially in academia. However, a few experts confirmed the existence of certain ethics procedures, especially in the NGO sector. These procedures or even committees reflect only on the internal institutional context, which cannot be considered alone in the ERC process and without being nationally accepted. For a well-functioning ERC system, most of the experts voiced the need for two actions as follows: (1) establish an integrated NHR body that will develop and embed regulatory, technical, scientific, administrative and legal frameworks and (2) reform an approved ERC mandate based on this framework. Addressing both areas would make ERC more professionally effective, credible and representative of all health disciplines and stakeholders based on solid guidelines.

### National health research policy in Palestine

The findings revealed that one of the most prominent pitfalls of the NHRP is clearly the absence of a formulated national HR policy or strategy. Meanwhile, there is a consensus only on the availability of internal policies for HR within some health institutions. The responses for NHRP availability were as follows: (1) the majority voiced “*absence of policy or strategy governing HR*”; (2) particular respondents said “*there are certain policies, plans or guidelines*”, others described existing policies as old and many declared they were not applied, while few echoed that the national health strategy addresses HRS in its draft; and (3) a very limited number of experts did not know about NHRP availability.

Additional file 1: Table S4 presents the experts’ perceptions concerning the reasons underlying the absence of NHRP as well as insights on what would facilitate the building of an effective NHRP. Some experts depicted the NHRP as one of the most prominent HRS problems. Cross-sectorial responses were converged. The most frequent and common reasons mentioned among all experts were:

1. The lack of a strategic vision for HRS, governance and leadership weakness, and lack of an organised body
2. Low awareness and knowledge about HRS
3. The scarcity of resources, the fragility of C&C, and unconstructive competitiveness and duplication in HR work among stakeholders
4. HR not embedded in HCS and not prioritised in the government agenda
5. Malpractice in the HR priority-setting
6. Donors’ influence and inconsistent agenda
7. The repercussions of political turmoil in Palestine

Building NHRP initially requires a political will to step vigorously towards establishing an integrated national governance body. This body would take the mandate of formulating NHRP and updating its agendas. The policy that is supposed to be formulated needs to include technical and legal guidelines. Further, HRS culture and awareness among policy-makers needs to be entrenched along with providing adequate resources to HRS. It is also important to urge the MOH and stakeholders to assume leading roles within HRS support from international NGOs. Moreover, forming a national health policy forum is needed to build, advance and monitor this policy. To achieve the above actions, experts proposed the presence of a national HR strategy, active roles of some players and bodies, existing partnerships, availability of expertise and institutional HR policies.

### The pattern of HR priorities

A consensus is reported on the non-existence of essential national HR priorities (ENHRPs). Instead, many denied that setting ENHRPs is systemically exercised, applied and complied with, institutionally and nationally. The responses were classified into three categories, namely (1) as “*there are no national ENHRPs, which are not institutionalised yet*”, which was the most frequent one, (2) the response “*yes, there are ENHRPs or formulating efforts*”, expressed less often or only nominally echoed among experts, and (3) “*do not know*” about ENHRPs, although very few answered this. Some experts stated that formulating ENHRPs and committing to them is a key problematic issue. Others pointed to the fact that current ENHRPs do not fully reflect the national needs and are influenced by a political agenda. Governmental experts emphasised that efforts to establish a directory for HR priorities had been carried out collaboratively by the MOH and PCHR in 2013, on top of a bilateral initiative in 2014 executed by the Ministry of Higher Education (MOHE), through the scientific research council (SRC) and the Islamic University. Moreover, NGO experts added that many of the documented and agreed ENHRPs were not being applied. They criticised the dissemination mechanism of these priorities among the stakeholders.

Regarding the alignment of ENHRPs with the HCS and essential national priorities, perceptions were very diverse. Some government experts stated that health policies were based on real needs determined through scientific methods and evidence. Likewise, a few academics and NGO experts declared that HR stemmed from national priorities concerning health, but without systematic approaches. Conversely, experts from the three sectors characterised HR in Palestine as ‘messy’ and ’fashionable’, not driven by national agendas, but responsive to donor agendas and individualised purposes. Many NGO experts and academics revealed that several public health projects and research are carried out by institutions, among them the PNIPH. These projects are partly driven by a national need but without significant impact due to different factors, namely (1) the influence of the donors and their inappropriate demands, (2) research for the purpose of evaluating programmes, and (3) a lack of stakeholder involvement. Eventually, the building of a national HRS body to address the challenges and to gear the donors towards national goals is the central priority. This common perception was a consensus among experts.

As Additional file 1 : Table S5 demonstrates, most of the common gaps related to ENHRPs setting were almost convergent. These gaps focus on the absence of a unified body and strategy as well as insufficient political concern in HR, where all current research efforts are dispersed. Moreover, the table reports technical gaps, malpractice of ENHRP setting, unsystematic exercise, a lack of updating, and misconduct in sharing and applying them. There is no national consensus regarding HR priorities because of conflicting research interests and agendas of stakeholders. Additional reported gaps were related to weak C&C, decision-making and research disconnection, as well as the scarcity of resources and an unsupportive environment. Insights were stated on how to make the ENHRPs process effective and reflective of society’s needs. Most notably, there is a need for political motivation to support the building of a national reference body leading a unified HR policy. Additionally, a systematic, active and participatory ENHRP setting and allocating essential resources, increasing the knowledge and professionalisation of ENHRPs exercises are essential. In addition to entrenching a strategic policy dialogue, enhancing C&C and communication mechanisms and regular oversight and guidelines on ENHRPs are also needed. Similarly, the donors’ agendas should be geared towards the national ENHRPs. All these proposals should be reinforced alongside previous HR priority initiatives and existing partnerships and bodies. Furthermore, the advantages of the LPHA should be maximised and used as a national exchange platform for ENHRPs.

Additional file 1: Table S6 reflects three ENHRP setting exercises, many participants in this study also took part in two other exercises involving all sectors. The first was held by the SRC of the MOHE in 2014 and the second was organised by the PNIPH and MOH in August 2017. This study represents the third exercise. ENHRPs identified by the first two exercises were mainly technical, while this study’s ENHRPs were more general. HCS areas were almost consistent among the three exercises, except for the current study’s government sector, which focused on the burden of medical referral costs. Non-communicable diseases, its determinants and causes were common ENHRP among the three exercises. This also applies to the nutrition area. Another agreement area among all experts, except the academics, was mental illnesses, disability and its services. The environmental areas were not also a priority for the academic sector. Infectious diseases have also been a research concern of all except the government experts. Importantly, the area of research policy does not receive priority status. Other miscellaneous HR areas varied, and included medical diagnosis and molecular and genetic diseases receiving the attention of the first two setting exercises. In the current study, the government experts outlined the causes of mortality and antibiotic resistance as a key research priority.

## Discussion

The overall findings indicated that stewardship within the Palestinian context is generally disappointing, not only in the HRS but also in the whole Palestinian HCS [26, 28], as in many developing countries [29]. The study found that a national governance structure for HRS is not clearly framed and defined yet. Different studies affirmed the absence of a formal NHRS [22, 34, 35]. Moreover, the functions of HRS governance and relationships among stakeholders are not well articulated nor well performed. In return, some HR institutions demonstrate good practice in terms of the established governance structure. Other consistent findings revealed that only four out of 10 countries had national HRS governance structures, whereas the overall research performance was poor with a critical deficit in stewardship function [17, 21].

As shown, the HRS architecture in Palestine is not clear-cut and to a large extent fragmented. In fact, it even appears to be uncertain regarding the functional and organisational flow of tasks and relationships. As HRS is complex [34], several national bodies were identified to lead HRS in Palestine both bilaterally or unilaterally, whereas the performance of these bodies leadership are unsatisfactory. In the current HRS map, the MOH, alongside with the three bodies PNIPH, PCHR, and SRC, seem to be those currently leading HRS, but not in a harmonised and synergic manner. The suitability of PNIPH to lead HRS remains controversial, since it is a project-based initiative formed via an agreement between the government, WHO and a Norwegian donor, and geographically not well represented [36]. In contrast to the known international standards, Palestinian universities and some NGOs and national agencies are HR producers, while the government is supposed to be only an HR user, as two studies revealed [37, 38]. HRS capacity in Palestine, while still weak, is present mainly in academia and NGO sectors [37, 39]. Importantly, this study found a wide discrepancy of perceptions concerning the functions and capacity of these institutions to act as a governance body. Because HRS governance is a collective and conjoint responsibility and cannot fall under one leadership, HRS entities require substantial reshaping and a harmonisation of their efforts to be comprehensively placed into a unified national perspective [6, 40]. This could be ensured by a collaborative strategic governance framework as well as very clear, well-negotiated definitions and descriptions of the roles of each actor [4, 41].

Two dimensions of governance challenges impede the establishment of a coherent HRS, namely (1) national and (2) structural and technical challenges. Nationally, disagreement on HRS visions dispersed the efforts and created parallel bodies with autonomous performance and significant inefficiency in using available resources. Furthermore, a lack of sovereignty over national resources and political instability caused by the Israeli occupation and intra-Palestinian division remain a key national challenge. The key features of the occupation affecting not only HRS, but also all governance sectors, are the closure of the international crossings and geographical segregation, inclduing blockage of the GS or checkpoints in the WB, which constrain the freedom of movement of patients, delegations and researchers, as well as the entry of goods [42–44]. Other effects are the excessive use of force, settlement expansion, illegal exploitation of natural resources, destruction of institutions’ and private property, and violation of international humanitarian and human rights laws affecting the social and economic conditions of the people [26, 45]. The intra-Palestinian division has affected the unanimity of Palestinian decisions and the institutional structures, leading to a severe decline in services and reduced wages of public servants due to tensions between the authorities in the WB and GS [46]. Recently, a reconciliation agreement was signed between the Palestinian parties [47], and this political shift may resuscitate the development of all sectors, and in articular the HCS and HRS.

The overwhelming technical and structural challenges facing the HRS are that concepts and practice are not fully entrenched in the health sector, as previously evidenced [48], a lack of leadership, accountability, monitoring and evaluation, regulated policy and C&C. This provides two indications:

1. HRS governance is individualistic and non-complementary.
2. Scientific research and HR are not on the government’s core agenda, since neither gets sufficient political attention.

Most of these findings are consistent with previous studies [7, 17, 21, 22, 32, 35, 37, 38], albeit revealing different gaps, most notably the lack of a conducive research environment and poor overall research performance, which is due to critical deficits in system stewardship, governance and infrastructure, lack of strategies, and political transitions. It is important to address these gaps while working on HRS strengthening and developing strategies or allocating resources [49]. It is expected that donors should work towards a unified HR agenda, since internal challenges and the lack of a unified vision concerning HRS repeatedly cause diverse and negative influence of donors on HRS [13], preventing the system from gearing its priorities appropriately [50]. This paper argues that the abovementioned gaps impede any serious actions towards restructuring HRS governance to reflect the national priorities.

Based on that, many studies coincide with this study’s recommendations of how to address these gaps [17, 21, 22, 24, 35, 37, 39]. The emphasis on the importance of political commitment towards the creation of a unified and clearly structured governance body embracing a legislative and organisational framework under an advisory board is essential. It is suggested that such a body should hold three assignments. Initially, to embed HRS values and the concept of stewardship into HCS and to develop an effective NHR strategy that includes instrumental policies. Subsequently, to establish a regular and needs-driven ENHRPs mechanism that involves all stakeholders. Finally, to promote the consolidated C&C and divide the roles of actors, as well as to exploit the existing efforts and opportunities.

This study noted that C&C for HRS is currently at a low level of performance. Its findings of a considerably fragmented C&C concur with previous studies [21, 22, 37]. Experts described C&C as being fragile, unsatisfactory and vague, with currently limited relationships and performance based on personal interests. Strikingly, one study refuted these findings, revealing that the international collaboration in research is evidently growing in Palestine [39]. Locally, it is recognised that C&C is a real challenge not only in HRS but also in HCS [26]. As in the governance part, the current poor C&C status of HRS is an inevitable reflection of the absence of a policy framework regulating the roles and responsibilities. Likewise, the lack of partnerships and teamwork is a key organisational gap. Another technical gap that contributed to poor C&C is the influence of donor agendas on HR [50]. All this leads to HRS work duplication and inconsistency of agendas. Additionally, there is the scarcity of resources and a disassociation between the decision-making and research levels [51]. These gaps create difficulties in data flow and knowledge sharing among HRS stakeholders [52]. Again, the political obstacles, whether induced by the Israeli occupation or the intra-Palestinian division, remain the main challenges for HRS development [39, 53] and clearly caused a structural and functional breakdown in the national institutions and relations. Thus, ending the occupation can unleash the Palestinian HCS, particularly HRS, and restore its full potential and capacity [44]. Additionally, unifying these institutions under a clear reference authority [54] is the nucleus for adopting the C&C model of COHRED, which calls for establishing well-synergised mechanisms for better HRS [55]. Regarding ERC in Palestine, there is a common perspective that ERC is weak with unpersuasive performance. Palestine is no real exception here, as different MER countries have insufficient ethical review and assessment capacity [56]. Nationally, so far, ERC has not been given much attention, although many Arab countries have recently started doing so [57, 58]. The Helsinki committee which is deemed as the only ERC national board is established in 1988. It constitutes various experts and academics mandated to assess the ethical aspects of HR. This committee is affiliated to the PHRC, while its political and legal ties with the MOH still need to be legally institutionalised. As ERC is structurally lacking, it is striking that the geographical work scope of this non-institutionalised committee is limited to review research in the GS, while this committee seldom scrutinises HR submitted from the WB. There is an urgent necessity to advance its professional performance and to make it more geographically representative.

Other flaws of the ethics committee are the unavailability of an ethical and legal national framework due to governance deformities and, consequently, a lack of guidelines and standards under the umbrella of the existing research ethics international guidelines [59] at the national level. A comparative study reported many differences to international guidelines in ethical practices in the MER [60]. However, certain institutions have institutional ERC or Institutional Review Boards or particular ERC procedures, notably in academia and some local and international NGOs. This study, along with other relevant studies, emphasises the importance of improving the efficiency of ERC [57, 58, 61] by founding a unified HRS. This would include an accountable and appropriate national REC board; one of its components is a regulatory, technical, scientific and legal framework aligned with international guidelines. Furthermore, efforts regarding the institutional ERC and capacities of professionals and researchers need to be enhanced. This can be realised through political decisions and guidance as well as the enactment of national legislation. Interestingly, ERC was not essentially addressed in the articles of the Palestinian Public Health Act or even in the MOH and PNIPH strategies; only regulations for the health professions, medications oversight, and healthcare improvement have been tackled [62, 63].

For NHRP, the findings show that a policy devoted to HR in Palestine virtually does not exist. In fact, only two out of ten countries in the region have dedicated NHRPs [22, 51]. There is a belief that absence of NHRP is a hindering factor for strengthening the HRS, together with the governance pitfall. On the other hand, as many experts affirmed, there are institutional HR policies organising research work. The Palestinian National Health Strategy for the years 2017–2022 [62] stated HR as peripheral, meaning that HR is not inherently a core component of this strategy. The reasons behind the absence of this policy are poor insight into the necessity of creating a strategic HRS vision as a basic component of the Palestinian HCS, low awareness of a HRS culture, and deficit of resources [21, 22, 24, 38, 51], while other less important sectors have the biggest share of the state’s public budgets. Furthermore, HR is not on the government’s agenda. Concerning the C&C, inappropriate collaboration and unhelpful competition as well as work duplication hinder efforts to build a unified NHRP. Likewise, misconducting ENHRPs makes the HR activities ill-directed and also restricts any strategic move to give precedence to designing an HRS regulatory framework. Finally, as delineated earlier in HRS governance, is the impact of politics, primarily the disintegration of the political and social system, on top of the donors’ imposition of their agendas at the expense of the national needs. In fact, it is of paramount importance to create an NHRP framework, which is a keystone of an effective NHRS [6]. As many experts revealed, challenges related to NHRP can be tackled through unwavering political and sustained financial support under the inclusive regulatory body and policy framework supervising the implementation and evaluation of this policy. This policy comprises a set of mechanisms and guidelines taking into consideration all HRS components [6, 19, 64]. Concurrently, the culture of HRS needs to be enhanced, and the existing strategies and bodies need to be re-employed to build this policy synergistically.

As far as ENHRPs are concerned, it is noticed that the exercise of HR priority-setting in Palestine is growing. This does not necessarily provide the agreed national HR priorities that Palestine lacks [51]. Some studies emphasised that there have been no previous priority-setting exercises in health policy and systems research in MER [65], with only three countries in the region having set national HR priorities [22]. Three important domestic exercises for HR priority-setting have been reported, in addition to other bilateral or multilateral institutional HR priority workshops. The first exercise was initiated by the MOHE with the PHRC in 2014 and resulted in the production of research priority manuals for all disciplines, including health. However, this exercise was limited to Gaza during the period of intra-Palestinian division; therefore, this exercise cannot be scaled-up unless it has national agreement and involvement, political adoption and a follow-up. The second was carried out in the WB, initiated by WHO via bilateral cooperation with the PNIPH. This study constitutes the third attempt, building on the previous two exercises and offering a common ground with them. Certainly, this study views these attempts as an essential step leading to further progress, although these attempts largely do not reflect the societal needs in the area of HRS.

Additionally, there are various gaps concerning prioritisation, mostly the lack of political power and its influence by social, political and environmental factors to meet specific interests, be they the government’s, the donors’ or personal [4]. Furthermore, a deficiency in knowledge and expertise is observed where these exercises are not practiced systematically in an integrated national perspective. Further, the issue of stakeholders’ compliance to the outputs of these prioritisation exercises, along with the scarcity of resources are problematic. The findings of inappropriateness in the application of stated ENHRPs and also improper dissemination agree with relevant research and are therefore considered as areas with a critical gap. For the proper ENHRPs setting, it is necessary to build on what has been achieved locally and to institutionalise exercises in a dynamic, inclusive and systematic approach [23]. Actions are needed, including obtaining political commitment, a regulatory body and national consensus on proper approaches of priority-setting [66]. These three prerequisite actions could ensure agreed ENHRPs and a good steering of the donors’ agendas. These actions could also form a strengthening pathway to develop all other HRS components. Developing them would mean providing the required resources and carrying out training to expand the knowledge and expertise of experts in ENHRP setting, encouraging the strategic dialogue and linkage between decision-makers and researchers, and adopting viable monitoring and updated mechanisms in prioritisation, guaranteeing that ENHRPs are disseminated appropriately among all parties [23, 67]. Additionally, the previous and current exercises and the existence of PNIPH and LPHA need to be developed and well exploited.

Through a comparison of the three HR priority-setting exercises implemented in Palestine, three thematic areas were identified according to frequency and ranking. The most important priorities to be addressed by HRS are the areas of health governance, financing and policy. These findings closely intersect with a local study which found that these areas are the main concern of ENHRPs [68]. Other regional research agrees that financing and workforce are priorities [65]. Further common ENHRPs are non-communicable and communicable diseases, nutritional conditions, disability and environmental issues; these areas form the major burden and causes of death and are the most affected by the escalation of instability and crises in the region [69–71]. The priorities of this study intersected those in Yemen and Oman, and agreed with priorities covered by LPHA in its research series [22, 72]. The area of medical diagnosis and genetic and molecular diseases was less frequently mentioned, meaning that it received low research priority. Nevertheless, two studies revealed a local discrepancy in priorities, indicating that the area of medical diagnosis and genetic-molecular disease had a high HR priority, while it was graded the seventh rank of the total HR publications in Palestine; this research area has been on top of Lebanon's HR priorities [22, 39].

Our study has four main strengths. (1) It is the first participatory study examining three important HRS components in Palestine, while this subject is inadequately investigated in the MER. (2) The participants and stakeholders were very diverse, including policy-makers, academia, experts, professionals, the private sector, and local and international NGOs. (3) Using mixed qualitative instruments was helpful for getting high trustworthiness of perceptions. (4) The purpose of the study was to generate insights to boost the three components of HRS, namely governance, policy and priority, and forms part of a larger investigation project that will lead to a comprehensive strengthening of the perspectives for the Palestinian HRS.

The study limitations were as follows: (1) A great paucity of relevant literature, reports and data on the subject, whether local or regional, thus not allowing meaningful comparative synthetic analyses and discussions, and making it impossible to use quantitative tools in analysing the HRS in Palestine. (2) Some time-constraints in questioning more participants and targeting of additional relevant institutions to determine all opinions, suggestions and views. (3) As other studies revealed (AlKhaldi et al., 2018, in press), field obstacles to the freedom of movement of the research team as a result of the geographical segregation and closure of security checkpoints. (4) The signing of the reconciliation agreement between the Palestinian political factions in October this year is likely to generate a positive political transformation that may affect some of the study findings, especially those related to the impact of internal political factors on the HRS and the HCS in Palestine.

## Conclusion

Attention to HRS functions is mounting, and there is a consensus that strengthening this system is imperative, especially in developing countries like Palestine. A well-functioning HRS is an inevitable reflection on an appropriate visionary management and policy. Therefore, the study provides a valuable snapshot of the three most important stewardship functions, attempting to understand them, to determine the obstacles and to generate solutions for a national well-performing HRS. The study primarily emphasises the importance of understanding the experts’ conceptual pattern of the three important functions, which is a basic demand in system analysis towards strengthening HRS. The importance of the study lies in its three dimensions. (1) Locally, it is the prominent research addressing this subject. (2) It contributes to filling a knowledge gap in the region. (3) It corresponds to international calls, notably by WHO and COHRED, encouraging countries to analyse their HRSs in order to boost national development.

The study found that the three stewardship functions are still not performing as they should. A structural HRS governance framework is missing; most of the HR activities are scattered and uncoordinated. Despite limited demonstrated good practices, the process of ERC is still weak due to the lack of an agreed national committee, lack of procedural quality, and non-compliance with ethics guidelines. Indeed, a functioning HRS cannot exist without a strategic national operational policy and regulatory mechanisms, which are lacking in Palestine. However, the exercises of prioritisation appear to be evolving despite the deficiencies, lack of consensus and low levels of knowledge and experience. It is noticed that the lack of political pledge, resources and capacity support, the absence of a national unified body, and the effects of the political conditions are the key factors impeding the strengthening of the HRS stewardship functions in Palestine.

In order to cover this subject fully, further empirical research is needed to explore the more evident institutional HR operations related to the three functions, as well as to examine the applicability of the HRS functions and its compliance with international approaches, models and guidelines.

There is an imperative need to initiate serious efforts to develop a national HRS in Palestine through focusing on strengthening the three functions. Initially, the attention of decision-makers in the various sectors should be drawn by informing them of these facts and obtaining political commitment and more mobilisation through a strategic policy dialogue. This dialogue shall involve all stakeholders to establish national consensus and agreed actions on three tracks towards enabling the three functions of the system. First, the importance of founding a unified national HRS body – the MOH is likely to be given the lead mandate to orchestrate this body regarding stewardship, resources mobilisation and regulation. The PNIPH could be that body – it was authorised by the state last year – but only after redeveloping it to become more representative and well-institutionalised nationally. Secondly, the necessity to start the formulation of a national policy for HRS through this body. This policy needs to comprise a technical, scientific, administrative and legal framework to ensure that the three HRS functions are appropriately working. More importantly, there is a need to reform the existing ERC for it to become a national and integrated professional committee that adopts international standards and has precise and clear procedures in the ethics process. Third, such a policy could essentially address the exercises of ENHRPs setting that need to be reviewed and combine all implemented exercises under a unified national entity. This is necessary in order to ensure a national consensus, comprising an inclusive involvement, systematic prioritisation, priority–needs matching, and well-disseminated priorities with a follow-up on their application. Additionally, raising knowledge and expertise concerning this exercise among stakeholders is essential.

These proposals constitute an important roadmap that could inspire all stakeholders to move forward. In fact, enabling the stewardship functions is a fundamental move that would lead to great benefit to the state authorities, who could take the mandate to regulate all HRS activities with unwavering support and utilise the outputs from HR. Other key stakeholders, such as academia, NGOs and the private sector, are also required to involve themselves actively in terms of HRS assignments, whether by funding, production or use. This should be realised through a well-shaped and coherent HRS framework where the roles are defined and coordinated, the operational policy is formulated and unified, and priorities are exercised systematically.

Therefore, ensuring the implementation of these strategic proposals even in a country like Palestine, with all its difficulties, can give a precious opportunity towards strengthening these system functions. This would encourage the Palestinian institutions to produce meaningful knowledge and useful evidence to be utilised for the optimal use of existing resources, improving the performance of the Palestinian HCS, and thus promoting the health of the people.

## Additional file

Additional file 1: **Table S1.** Responses on health research (HR) governance challenges and improvement opportunities. **Table S2.** Responses on HR related to coordination and cooperation status, gaps and improvements. **Table S3.** Responses on the status of HR ethical review and clearance. **Table S4.** Responses on the status of HR policy, gaps and improvements. **Table S5.** Responses on the pattern of HR priorities, gaps and improvements. **Table S6.** Comparison between HR priorities defined in this study and those defined by Palestinian National Institute of Public Health in August 2017. (DOCX 76 kb)

## Abbreviations

C&C: coordination and cooperation
ENHRPs: Essential National HR Priorities
ERC: ethical review and clearance
FGDs: focus group discussions
GS: Gaza Strip
HCS: healthcare system
HR: health research
HRS: health research system
IDIs: in-depth interviews
LPHA: Lancet Palestinian Health Alliance
MER: Middle Eastern Region
MOH: Ministry of Health
MOHE: Ministry of Higher Education
NGOs: non-governmental organizations
NHRP: national health research policy
PCHR: Palestinian Council for Health Research
PNIPH: Palestinian National Institute of Public Health
SRC: Scientific Research Council
WB: West Bank
