# Supplementary material for: The Palestinian health research system: who orchestrates the system, how and based on what? A qualitative assessment
Source: Health Res Policy Syst. 2018 Jul 31;16:69. doi: 10.1186/s12961-018-0347-4 (PMC6069718; doi:10.1186/s12961-018-0347-4)
Supplement: Supplementary file 1 — Table S1. Responses on health research (HR) governance challenges and improvement opportunities. Table S2. Responses on HR related to coordination and cooperation status, gaps and improvements. Table S3. Responses on the status of HR ethical review and clearance. Table S4. Responses on the status of HR policy, gaps and improvements. Table S5. Responses on the pattern of HR priorities, gaps and improvements. Table S6. Comparison between HR priorities defined in this study and those defined by Palestinian National Institute of Public Health in August 2017. (DOCX 76 kb) [file 12961_2018_347_MOESM1_ESM.docx]

**Palestinian health research system: who orchestrates the system, how and based on what? A qualitative assessment**

**Additional file 1**

**Table S1: Responses on HR governance challenges and improvement opportunities**

**Table S2: Responses on HR related to coordination and cooperation (C&C) status, gaps, and improvements**

**Table S3: Responses on the status of HR ethical review and clearance (ERC)**

**Table S4: Responses on the status of HR policy (HRP), gaps, and improvements**

**Table S5: Responses on the pattern of ENHR priorities (ENHRPs), gaps, and improvements**

**Table S6: Comparison between ENHRPs defined in this study and HRPs defined by PNIPH in August 2017**

**Table S1: Responses of HR governance challenges and improvement opportunities**

| **Governance** | | | |
| --- | --- | --- | --- |
| **Theme**  **Sector** | **Theme 1: Challenges** | **Theme 2: Improvements** | **Theme 3: Opportunities** |
| **Gov.** | - Non-aligned vision and work fragmentation - The deficit of legislation framework and laws - Unclear political reference to lead research which is driven by donors mostly for short-term - Lack of national unified HR policy - The unstructured and unclear framework - HR is not institutionalized and sustainable - HR actions are malpractice due to the absence of collective regulating body - HR is unconsidered politically and unvalued - Lack of accountability and HR awareness - Individualism rather than collectivism - The economic crisis, resources scarcity, the political situation - Research non-linked with our life activities and institutional processes - HR regarded into NHS but yet is not implemented - Ministerial inauguration changes - Research duplication and bodies multiplicity - No research M&E | - HR national committees - National governance entity by MOH - Intergovernmental governance and C&C, sufficient-secured fund and staff - Certain vision to formulate a national policy leads to a national body - The regular setting of HR priorities by a collective body - Develop a legislation framework regulates all HR actions - Promote C&C - Gov. and MOH should take the responsibility of leading HRS and allocate 5% of its budget for HR - System reform is needed - MOHE is required to cultivate inherently research philosophy in the education system | - PNIPH-PCBS to take the lead - Public health law - HR department at MOH and universities, and MOH-academia inclusive partnership - Availability of excellence centers - MOH mandate and responsibility - Exploit PMC and PCHR existence - Qualified enough alumni and experts - Previous initiatives to build on - The general believes that HR is a benefit - HR is regarded in the Palestinian NHS |
| **Acad.** | - The multiplicity of entities and fragmented existing national councils or committees - Transparency and EBD are missing in the health sector - HR is not a core part and priority in the HCS - Lack of state independence and stability - Political unwillingness nationally and institutionally - Low state capacity and occupations effects - Institutions autonomy and centralization - Lack of qualified staff and effective body - Lack of sovereignty and structural problems - Limited role of MOH and Universities - Resources scarcity and agreed priorities are missed - Low research credibility and quality - The fragility of C&C and partnership - Lack of enabling research environment - Individual effort-oriented | - Research national council or committee, led by MOH, involving all stakeholders to set strategies and priorities - the This research needs to be a centric and inclusive adopted by the state to respond to social needs and coordinate HR actions - National policy and brainstorming workshop addresses the issues of HRS - Activate the role of PCHR and HRD at MOH for proper research facilitation and translation - Delineate the roles of HRS players - Permanent annual budget and commitment - More an official attention to HR - More C&C among academic thinkers, health providers, and international funders | - State institutions are under developing - Mounting importance of HR - A key pillar of the country infrastructure - PNIPH as a platform and reference - Activate PCHR and HRD at MOH - HR is a pure academic role - MOH leading role of national priorities setting |
| **NGOs** | - Indistinct role of academia to lead initiatives - Research-externally solicited and controlled - Individualism purposes and scattered efforts - HRS is not embedded in HCS, lack of leadership, policies, resources, and community interest - Structural organizational dilemma - HR not a prioritized and undesirable concept by gov. - Centralization and bureaucratic political system - The absence of national HR policy and poor EBD - The disconnection between researchers and policy making - General social, political, and economic factors - The inefficiency of MOH management, resources mal-distributed, and its role is a vague mandate, provider or regulator, to lead HR - Paradoxical of stakeholders’ interests - Fund deficit in gov. and academia to HR - Bodies multiplicity and low research awareness - Being HR confined in some individuals prevent to not practiced widely due to no system - Limitations of time and management support - The weak legal framework in academia and often does not exist in NGOs - Chaos status and scattered-reactive HR initiatives - All health actors are not represented in decision making - No governance causes lack of research quality | - National HR supervisory committee to set the actual priorities - All stakeholders should agree on national and institutional policy - HR governance should be non-centralized - A clear strategic structure is needed to guide HR efforts, identify its priorities and translate evidence into actions - Support the young researchers and adopt a multidisciplinary research - An inclusive national HR association, combine gov., academia, NGO and private - Commitment and construct HR policy and activate a collective body with a platform for HR priorities setting - The state, MOH, should lead and involve all actors in a national body - Allocating sufficient resources - Establish HR legal framework to validate research validity and quality - More support from the international agencies | - PNIPH presence and other bodies, its governing body, and trusted capacities, although it is criticised - Plenty of qualified postgraduates and researchers - Collaborative ties among WHO, MOH, and UNRWA - MOH governance structure - A belief that HR is a strong weapon - Identified health priorities - Existing institutional strategic structures and internal policies - MOH-academia partnership - MOH-PNIPH partnership - Existence of Supreme health council mandate and working under WHO umbrella - Pioneer and active role of some universities to cooperate - Presence of HSWG, HRC - Agreed COC across NGOs - The assistive role of UN agencies |

**Table S2: Responses on HR related to coordination and cooperation (C&C) status, gaps, and improvements**

| **HR related to C&C** | | | |
| --- | --- | --- | --- |
| **Theme**  **Sector** | **Theme 1: the status of C&C** | **Theme 2: C&C Gaps** | **Theme 3: C&C improvement ways** |
| **Gov.** | - Very fragile, independent, fragmented and random efforts weakening the stakeholder’s contributions - Weak, poor, lack of international exchange programs, - Not well established but PNIPH plays C&C role - Competitive more than cooperative or integrative, especially between universities - Still passive without any effective and institutionalized ties, but some good individual connections exist - Lack of C&C with some limited links among players - Relationships are poor and at the lowest level - There is no integration, all are working independently - Good between gov. departments, there is a regular C&C among ministries through advisory committees - Individualistic swinging based on interests - There is still a gap between relevant parties except for respective bilateral collaborations e.g. WHO and MOH - Biased relationships in research actions - The worst element in the health sector in general - Fluctuated due to changing executives inauguration | - The absence of a collective body or integral system - Each institution does a great effort at the micro institutional level, but the collective is missing - C&C is not established whereas completely personal relationships - No national and institutional policies for managing actions - Political instability and HR is not a priority, - Lack of awareness and resources - No spirit of collective teamwork - Non-supportive attitude - Fund and time limitations - Knowledge is not disseminated among producers and decisions makers, where they are disconnected - Data for research are not completely shared and accurate | - Develop a collective regulatory body to attain better communication and interaction, - Consolidate C&C mechanism with all stakeholders, mainly academia and gov. - Technical advisory committee to monitor HR priorities and allocate resources - Make real partnerships, recognize and divide the roles based on a common vision to prevent a repetition - A need for serious and cooperative dialogue between all stakeholder’s - A unified platform for exchange - Promote the sense of we all complement each other, get rid of donor control, financial independence, and using technology to enable connections - Achieve the sectors integration - MOH role in promoting the HR culture - Define the stakeholder's research roles - PNIPH is a key player to improve HRS - Collaborative capacity programs to develop researchers competencies |
| **Acad.** | - C&C is poor, very weak and literally paralyzed - There is no C&C, which is the only personal basis - A complete disconnection between organizations which is mainly individualistic and selective - Differentiated visions, driven by personal relationships and interests - We are not cooperative with each other - Weak connection between academia and gov. - Most often, there is C&C, but some individual - No agreement on the priority among all stakeholders - Recessive C&C between MOH and academia, which is not either organized not effective based on reactions - Not fully satisfied but there are some partnerships - No clear relationships, policymakers rely on internal reports to take decisions - No system, commonly successful individual attempts - Generally dissatisfied - Weak C&C which deprives Palestine of good opportunities in successful in building external collaborations while failed national or locally - Most of the HR efforts are unorganized | - The MOH apathy to invest in schools research production - Most of the public sector research is driven and controlled by external bodies such as WHO, EU...etc. - Because HR is non-institutionalized without a C&C strategy - Individual priorities and interests - Non-integrated institutional work - Mistrust between health institutions - Duplications and disconnection - Government bureaucratic system - Weak C&C even in intergovernmental departments and nongovernmental institutions - Duplication of research activities - Most relations are individual and personal connections - Political internal division affect negatively on relationships - Most of the universities are private who keep working independently - C&C subject to personal networks and funding parties | - Develop agreed agendas, vision, and policy with a focus on the research types and quality - C&C mechanisms and strategic partnership (gov., academia, and NGOs) - More investment in the annual meeting of Lancet Palestinian Health Alliance (LPHA) - A platform for meeting and discussions - Exploit the plenty of INGOs in building capacity and partnerships to implement advanced research and technology - Form a national research body or committee led by MOH involving all stakeholders to set strategies, needs, regulations, ways for knowledge sharing and develop staff competencies - A clear and inclusive C&C policy updated regularly and connect HR funders, producers, and users - incentivize researchers and reduce teaching loads - Less bureaucracy and more supportive environment - Public-private partnership (PPP) along with gov.-academic-NGO partnerships and academic unified association - Multidisciplinary research is crucial |
| **NGO** | - There is no connection - Scattered and non-linked agendas - Lack of communication between HR producers and users hinders evidence translation - Good relationship but not integrated - A key weakness to be investigated and treated - Relationships are weak and competitive instead of collaboration - Very weak, interrupted and disorganized - There is no C&C between all entities - Competitive rather than complimentary - C&C is seasonal and vague - Poor C&C based on destructive competition - Quite good especially at INGOs level, but between providers and academia is regressive and PNIPH is too far from others - Moody, temporary and competitive - Weakest pillar in the HCS at all - Lack of knowledge accessibility and sharing | - Research is unsystematic and personal-performed - Stakeholders reliance on external sources with uncommon visions - Difficult knowledge accessibility and sharing among stakeholders - Lack of organization and communication between policymakers and scientists - All HR activities are occasional rather than systematic - Research conducted just for programs evaluation - The absence of organizing body - Public-private partnerships are lost - Lack of faith in C&C and the spirit of teamwork - Overall worsening situations | - Finding an integrated system to make the parties more cooperative, and research outputs more shared and applied - A united HR strategy, each party takes a certain role - All stakeholders must collaborate in identifying the health priorities - Policymakers need to motivate creating collaborations, increase fund, and set HR priorities - Local C&C mechanisms between policymakers and academics such Lancet forum as an excellent initiative to be exploited - A need for MOH and MOHE leading role - Create a network and promote the teamwork in a complementary approach - C&C agreements and sectoral partnerships - Established agreed and C&C term of reference - Widening the awareness on HRS |

**Table S3: Wide-ranging quotes from experts on the Ethical Review and Clearance (ERC)**

| **Expert No. Key quotes** | |
| --- | --- |
| “There are ethical reviews such as HELSINKI committee, equally, there are committees at the institutions level but they are not well structured and need to be organized and developed” | **Gov. Exp. 2** |
| “In MOHE department, research that targeting students at schools should get an informed consent, especially in lab tests, from the parents, students themselves, and the officers. There are no obvious and known processes where our focus basically to get this consent for questionnaires on behaviors in general” | **Gov. Exp. 4** |
| “PCHR was established to be a regulatory body in Palestine including the ethical committee to supervise and appraise the ethical aspects of submitted research. Our research department at MOH is responsible for protecting studies subjects from harmful interventions” | **Gov. Exp. 6** |
| “The ethical review process is nominal, we do review academically. The weakness that is not nationally and legally supported. HELSINKI committee is not officially active and has no specialization for all health research. E.g. in the Al-Quds University, we do not have a committee to review the research; we do this process academically” | **Aca. Exp. 1** |
| “There is no governance at all for research in Palestine; It all about individual efforts. And there is no coordination between organizations, regulatory body, regulation of ethical standards, neither scientific nor strategic point of view” | **Aca. Exp. 3** |
| Nationally no, but there are institutional ethical committees, even at my university, which is not reflected the national level. | **Aca. Exp. 4** |
| “We have ethical committees, of which at university and one in MOH which is weak. Plus "Helsinki" in GS, and local committees in WB, but their review quality and procedures are questionable, but at least, we have some with the lack of experience. E.g., we are measuring a clinical trial on some medications; we do not know the right department at MOH, who charge this procedure” | **Aca. Exp. 6** |
| “On the level of the university, we have a committee called Ethical Committee, hence, there is a good progress regarding the ethical review, but it lacks a clear structure” | **Aca. Exp. 10** |
| “To some extent, there is a clear policy dedicated to ethical arrangements in conducting health research under MOH duty” | **Aca. Exp. 11** |
| “Research proposals reviewed by HELSINKI, which has a slow process, are mostly not ethically harmful. In my university, we are about designing an IRB for students' researchers. A need for a national ERC supported officially by the government” | **Aca. Exp. 12** |
| An ethical committee with clear reviewing process is the first step when a decision-making body established. Our experience in this component is still weak. We need national, regional, institutional ethical committees in academic institutions. | **Aca. Exp. 13** |
| “My knowledge about the ethical review is limited, whether they have any ethical committee. In UNRWA, we just have internal ethical procedures, so we just follow this” | **INGO Exp. 1** |
| “We do not have a national health committee, such HELSINKI in Gaza is not very well-known, and do not know if its functions performed efficiently” | **INGO Exp. 2** |
| “ERC is crucial for all research targeting human participants and seeking fund, to check the clinical trial protocol appropriateness, ERC is a part of MOHE, where needs further development, such awareness and educating researchers on research ethics” | **INGO Exp. 4** |
| “I am not convinced that the ethical issues in our country are well applied, and the ethical committee should follow the international rules” | **INGO Exp. 5** |
| “In UNICEF, we have criteria where we reviewing our research via regional committee not national or local. The review was before three or four years, and then we conducted this study. In that time, I did not know if there were any committee concerns in ethical reviews locally” | **INGO Exp. 11** |

**Table S4: Responses on the status of National Health Research policy (NHRP), gaps, and improvements**

| **NHRP** | | | |
| --- | --- | --- | --- |
| **Theme**  **Sector** | **Reasons of NHRP unavailability** | **Improvements** | **Opportunities** |
| **Gov.** | - The weakness of systematic governance structure - We are under emergencies, short-term planning - No official body, regulating rules, and code of ethics for HR - The absence of HCS needs, insufficient budget, conflicted agendas - HCS is emergency-based, to maintain staff wages and care continuity - Political-economic crises conflicted priorities, HR is not a priority, economic and resources crisis - HR culture and awareness is lacking - Lack of political attention, adoption, leadership, absence HR from the agendas - Lack of resources, HR staffing, and guiding protocols - Researchers competition and low HR credibility - C&C and organized HR efforts are missing - HR is not a gov. priority - Donors playing a major role in formulating policies - Lack of political stability - HCS structural vibrations due to the political-environmental turmoil | - Unified national organizing body to be integrated into HCS to regulate all HRS processes, mainly policies - A clear HR policy reflects the HR priorities and country’ needs and applies its results - Political willingness and vision - Affording budgets and fund - Rules enhancement and real sectorial policy dialogue - Acknowledging HR importance, hiring staff for HR especially community social researchers - MOH needs to build a policy promoted C&C among players and enable HR in their facilities - Increase awareness about HR and evidence translation among policymakers - Consistent and persistent HCS non-affected by politics factor - Active team to develop HRS pillars - Establish research national agendas updated by MOH and academia | - We have a good strategic health plan, some HR areas e.g. NCDs and MCH have good improvements - We are developing but still in a random and individualized way - MOH and WHO guiding role - PHRC to take governing lead and HRD at MOH to coordinate - Qualified enough of experts - HR is stated in the NHS |
| **Acad.** | - Insufficient culture and willingness to change the traditional paradigm, HR in NGOs sector depends on donors and not in its system, the biggest duty of HR on academia which considered it academic need not national, the absence of regulated guidelines - Donor-imposed agendas - The poor fund, resources, and budgets - MOH attitude is not HR-oriented unlike academia - Problematic due to stakeholders competition not C&C, political instability and a fragile economy - No governance and gov. decision and strategic trend to HR tackle health needs, weak academia role, and C&C, differed health priorities, and legislative bodies and rules are ineffective - Non-experienced policy makers - HCS is not research-oriented and HR is not a core component, a gap between stated plans and HR application, poor health system studies and lack of motivation - Political and power conflicts in decision making, lack C&C, fund and unified plan - Missing stated priorities and unplanned research - Institutional and personal independence, lack of officials attention - HR is not the main part of our plans, weak culture of HR and evaluating them - Policies may control the innovation spirit of researchers, misapplied, individually-driven not a system to avoid objectivity biased | - Allocated sufficient fund and upgrading HRS infrastructure, improve the culture and education curricula on HR, encourage decision makers to be research-oriented - Expanded meeting to create national agendas and reference well-regulated body to govern HR activities - Formulate a policy with deducted fund from central budget for HR based on society priorities - Stimulate academic role in putting pressure on DM level to formulate HR policy to be applied as well, more workforce investment and motivation on HR - Founding a gov. HR body and then a committee to inform evidence to DM - Frequent needs update, HR policy developed and updated regularly by all stakeholders with active sectorial C&C - Comprehensive policies and regular priories setting - Promote the concept of evaluating our HR and motivation for researchers and - No need to reform policies but set guidelines built on society needs to give researchers freedom and to guide them technically | - Gov. sector is in the most need for HR concept and its application, the biggest pioneering role of academia in HR - Existing institutional policies and research committees - A great academic potential to convince policymakers - WHO-PNIPH partnership and their attempts to set agreed HR priorities - Previous attempts by SRC |
| **NGOs** | - A key problematic issue of Palestinian HRS - A clear vision and well-designed system is missed but mostly HR are institutional-performed for their programmatic needs, community interest on HR - Inadequate resources and budgets - HR is not part of HCS, lack of researchers and leaders - Lack and non-agreed priorities due to financial and political tensions, interests and variability - Vague and weak role of MOH and PNIPH is not well-linked with all stakeholders - No spirit of teamwork, duplication, competition, and improper C&C and communication gap between sectors, policy and research groups - Malpractice in setting and applying HR priorities - Misconduct of HR publication and dissemination process - All HR is individualistic, not developmental run in a competitive way leads to improper conduction - No political commitment and inability of leadership and managerial roles - The absence of a collective governance body or policy - Lack of a strategic vision for sustainable health development - Human value is under-valued - HR is a new culture and concept - Documented policies for propaganda without real application - Lack of policy leads to the accumulation of unused HR and kill the enthusiasm among researchers - No rewarding and incentive system for the research community | - Regulated institutional HR policies, viable health policy forum, regular HR M&E - PNIPH policies reflect the national priorities but need to be developed by all stakeholders to be adopted - A clear HR structure is desperately needed - Political interest and commitment to HR development - An organized and clear HR framework and reprioritize regularly our HR areas - A body included an internal system or platform and involves all stakeholders to determine HR priorities and to follow up HR - National policies and strategies for HR - Activate the MOH mandate to lead a national body and involve all stakeholders under PNIPH framework - Partnerships with the local and international scientific community, political tensions should be separated from the development process, a collective platform, academia-state C&C, reallocate resources for HR and budgets for academia to enhance research-based education and mutual programs - A legal HR framework for technical and ethical guidance, quality, validity, and utilization - More role and support from international agencies and NGOs - HR managing duties should be collaboratively divided among stakeholders | - Previous attempts by MOH to set national health priorities, - The existence of PNIPH and its policies - We always refer MOH as a reference regulatory body of HR - The existence of the national council for HR which is inactive where NGOs are not represented in its board - INGOs and national NGOs interests and initiatives in sponsoring and using evidenced-knowledge production in their interventions - Palestine is better than some Arab countries on HR - The existence of health sector working group HSWG - HR policies stated in NHP 2011-2013 |

**Table S5: Responses on the pattern of HR priorities (HRPs), gaps, and improvements**

| **HRPs** | | | |
| --- | --- | --- | --- |
| **Theme**  **Sector** | **Gaps** | **Improvements** | **Opportunities** |
| **Gov.** | - Lack of HRPs setting exercise - Not often updated, applied, unsystematic, non-compliant - Insufficient studies generate knowledge - Conducted HR are mostly derived from personal and donor desires with few are met HRPs - HRS and HRPs are not a political priority - No organized system, non-agreed strategy or HRPs - HR productivity is messy and scattered - Lack of resources and fund - Conducted HR is mostly a response to an emergency condition - No accountability and transparency in sharing HRPs - The sluggish role of MOH and government towards HR in general - PNIPH is still not completely ready - Many HRPs attempts, affected by donors agendas, are not completely scientific process - A gap between policymakers and researchers | - Political commitment, regular setting, technical advising committee from all parties - Collective system integrated into HCS supervised by MOH to manage HRPs and more sectorial C&C - Trusted data, statistics, and evidence - Allocate enough resources - Recruit full-time researchers - Realistic and dynamic HRPs setting based on actual needs - Increase the HR culture and background of policymakers - Serious and real sectorial dialogue and teamwork to formulate systematically HRPs - Research exchange programs to raise experience and knowledge - HR policy ensuring good HRPs exercise, sharing, and application | - Previous attempts, PNIPH efforts in HR prioritization - HRPs have been set by SRC under MOH embracement - We have pioneered human resources and potentials |
| **Acad.** | - There are efforts reflect the academic viewpoint, not health providers - No national body and policy to lead HR - HR are produced for promotion and academic goals but not nationally-driven and planned to improve the society - Many unsystematic attempts were not identified HRPs in a scientific efficient way where all have produced a list of wishes - HR and PHRs are donor driven and influenced not nationally - HR in academia and NGOs are occasionally conducted for special purposes, e.g. evaluate their programs needs - Paradoxical individual interests on HRPs and lack of national consensus - Lack of C&C - A failure of HRPs application and compliance - HR is not the MOH priority with the absence of enabling environment - HR production and application gap - Centralization is unhelpful - A huge gap between improving public health services and HR | - Direct our HR though national strategies under the unified organized body to set a unified vision reflects the actual society needs - A regular HRPs exercise should be performed considering the global HRPs - Empower the infrastructure - Allocate generous financial and human resources - Promote learning organization approach - Improve the sectorial C&C - Avoid the health sector from any political issues - HR should be based on society’s needs - Unified governance regulating the body, e.g. a state council, includes academia and government to set national HRPs and policies - Policymakers and researchers involved in HRPs exercises - Reject the external aids if do not serve the national needs - SPHC-WHO and other partnerships should be promoted in HRPs setting | - Previous stated HRPs through PNIPH and other to build on - Hopefully, PNIPH could take this lead - Institutional HRPs guiding the national efforts - The initiative includes Palestine, 9 countries have formulated a relevant policy to HRPs in 2009 - HRPs guidebook made by IUG and MOHE - PNIPH became more focused on other important HRPs - SPHC-WHO previous HRPs exercise in 2013 - SRC-PCHR cooperation |
| **NGOs** | - HRPs are driven by institutions based on their own agendas - HR is not a priority of the government - Existed HRPs are not endorsed by decision makers and shared with researchers - HRPs exercise is not dynamic and regularly updated for - Donor agendas and politicized money that inconsistent with local needs - The absence of a collective body and strategic agreed vision - Lack of communication, C&C, and team working with stakeholders and prioritization malpractice - Contradicted NHPs which are affected by the financial and political circumstances - HR is defined and conducted based on individual interests with few are priorities-based - Inability to apply NHRPs due to the absence of managing system - HRPs is not long-term and changing priorities due to country emergency status and instability - Conducted HR are scattered and not fully compatible with priorities, where performed for certain purposes, e.g. programs evaluation needs and researchers preferences or self-career - Most of HR are duplicated and disorganized - MOH depends on their own reports when identifying the society needs while other providers and partners are existing | - An HR regulatory policy under a collective and well-organized body - HR should take into account the international guidelines - HRPs National consensus reflects our needs based on research evidence - Accurate and shared database for PHRs - HR topics should be matched with identified HRPs - A clear sectorial strategy to ensure C&C in HRPs setting and systematic M&E - Support from the international support to the local HR and encourage them to adopt the local needs - Develop the human resources capacities in determining NHRPs - Guidelines should be established to regulate NHRPs setting - A committee to set HRPs - HR must be effective, efficient and disseminated to decision making to fulfill the needs - Policymakers attention should be drawn to the importance of HRPs and appoint HR advisors to them - Education curriculum development to be research-oriented and needs-reflected - National political unity and vision and not to politicize the vital sectors - Founding research units across the institutions | - A new draft of HRPs conducted by PNIPH 2017 - UNFPA priorities reflect the population needs - National priorities are listed in the SNP of the MOH - UN agencies and MOH partnerships - The active role of academia in this regard - A need to support the efforts of PCHR   MAP-UK and ICPH partnership through LPHA |

**Table S6: Comparison among three national HRPs setting exercises, SCR’s manual, PNIPH workshop and the current study’s perceptions**

| **National setting exercises** | **SRC’s manual on HRPs, 2014** | **PNIPH workshop on HRPs, 2017** | **HRPs identified by study’s experts** | | |
| --- | --- | --- | --- | --- | --- |
| **Areas** |  |  | **Gov.** | **Acd.** | **NGOs** |
| **HCS** | Health financing, HIS, workforce capacities, education and medical accreditation, coordination, management system | Access, coverage, workforce, PHC, health financing, HIS | Cost of referral abroad | Governance, resources allocation, health economic, care quality | Financing and policy, accesses, workforce, care quality, efficiency |
| **MCH** | Healthcare and protocols evaluation, school health, nutrition, anemia, child obesity, FP, early detection of genetic disease | Maternal, PNC, FP, women’s’ health, vaccination, nutrition | Child Behaviour | MCH | MCH and youth |
| **NCDs** | Causes and risk factors, assessing prevention-promotion, diagnosis and management, health care quality and providers performance | Preventive care, tobacco control, healthy lifestyle, cancer, CVDs, stroke, HTN, DM, determinants | NCDs, cancer | NCDs | NCDs, cancer, social determinants |
| **Nutrition** | Anaemia, providers roles, association with NCDs, food toxicity and pesticides, obesity | Anemia, vitamin deficiency, obesity | Nutrition, anemia | Nutrition, thalassemia |  |
| **Mental health** | Causes, addiction, suicide, prisoners, wounded and wars victims, care quality | Psychosis, stress-related, disability |  | Mental illnesses | Mental illnesses, disability |
| **Environmental health** | Water, air and soil, and diseases, wars remnants, industrial effects, medical waste management | Water quality, waterborne diseases, toxins, safety, traffic safety, buildings | Water and environmental health, RTA |  | Water and environmental health |
| **Infectious diseases** | Risk factors and causes, assessing of prevention-promotion programs and protocols, surveillance | Meningitis, leishmaniosis, foodborne diseases |  | Infectious diseases, NTDs | Infectious diseases |
| **Research policy** | Interdisciplinarity in basic, clinical and community sciences, excellence centers, evidence-based medicine, medical education, ethical and jurisprudence | HR capacity, accessing grants, publishing papers, data sharing, and analysis |  | Medical education |  |
| **Others** | Dental care, advancement of medical diagnostic methods, genetics and molecular biology, pharmaceuticals and natural plants use, medications financing and supplying |  | Mortality causes, antibiotic resistance | Osteoporosis, Genetics diseases, molecular biology, medical diagnoses | Socio-economic and political determinants |
